# Social media sharing, psychological distress, and student well-being: a PLS-SEM and fsQCA analysis of Chinese college students

**DOI:** 10.3389/fpsyg.2025.1554882

**Published:** 2025-07-23

**Authors:** Bo Shu, Zhigao Dong, Fang Su, Zheng Wang

**Affiliations:** ^1^School of Management, Jinan University, Guangzhou, China; ^2^Faculty of Health and Wellness, City University of Macau, Macau, Macao SAR, China; ^3^Department of Public Administration, Jiangxi Administration Institute, Nanchang, China

**Keywords:** social media sharing, psychological distress, students well-being, digital well-being theory, PLS-SEM, fsQCA

## Abstract

This study explores the relationship between social media sharing behavior and the mental health and well-being of college students. Based on the Digital Well-being Theory, we applied PLS-SEM and fsQCA to analyze data from 534 students across two time points. Results show that school-related (*β* = 0.198, *p* < 0.05) and social-related (*β* = 0.234, *p* < 0.05) social media sharing behaviors positively impact well-being, while work-related sharing (*β* = 0.247, *p* < 0.05), though also positive, can increase psychological distress. Psychological distress significantly reduces well-being and mediates the relationship between social media sharing and well-being. However, perceived support effectively moderates this effect, reducing the negative impact of psychological distress on well-being. FsQCA analysis identified multiple combinations of factors leading to higher well-being, emphasizing the importance of considering diverse influences (fsQCA coverage = 60.01%). The study provides practical insights for educators, policymakers, and students on using social media to promote mental health and well-being.

## 1 Introduction

In today’s digital era, social media has permeated all aspects of life, becoming a pivotal platform for daily communication, information sharing, and emotional expression (Baccarella et al., 2018; Bekalu et al., 2019; Beyens et al., 2020; Chen et al., 2022; Choi and Toma, 2014). The 2024 CNNIC report states that by June 2024, China had 1.078 billion internet users utilizing social media (China Internet Network Information Center [CNNIC], 2024), with college students being a prominent user group (Chen et al., 2022; Kim and Kim, 2017; Kim et al., 2011; Liu et al., 2023). Social media is a primary tool for interpersonal interaction and information acquisition for current college students (Jiang et al., 2023; Kim and Kim, 2017). College students, at a critical period of identity formation, value development, and socialization (Granieri et al., 2021; Kim, 2024; Lin and Zhan, 2024), have well-being, composed of positive emotions, connections with others, and life goals, which is vital for their development (Huta and Waterman, 2014). As a unique group transitioning from campus life to society, they face multiple challenges related to academics, social interactions, and work demands. These challenges often damage students’ mental health and can even lead to psychological distress. Psychological distress often manifests as negative emotions such as depression, anxiety, and stress (Man et al., 2025). Sharing on social media helps them exchange academic information, engage socially, understand job requirements, reduce uncertainty, and alleviate anxiety and depressive, significantly impacting their well-being (Büchi et al., 2019; Choi and Choung, 2021; Choi and Toma, 2014).

In recent years, a large number of studies have explored the impact of social media use frequency on students’ social behavior (Kim and Kim, 2017), learning attitudes, and psychological states (Büchi et al., 2019; Ellison et al., 2007). Moderate use of social media can help students improve their social skills (Khan, 2017) and avoid fear of missing out (Przybylski et al., 2013; Tandon et al., 2021), while excessive use may harm mental health. However, current research seldom analyzes how different types of sharing behaviors—such as school-related social media sharing (Watson et al., 2022), social-related social media sharing (Mao, 2014), and work-related social media sharing (Dantas et al., 2022; Schmidt et al., 2016)—individually or collectively influence students’ psychological distress and well-being (Zhu et al., 2024). Additionally, perceived support on social media is a crucial factor affecting students’ psychological states (Danielsen et al., 2010; Eagle et al., 2019; Grey et al., 2020).

This paper introduces the Digital Well-being Theory (DWT) as an analytical framework (Büchi, 2021), to comprehensively understand this phenomenon. This theory emphasizes how individuals’ behaviors and experiences in the digital environment influence their overall well-being (Büchi, 2021; Büchi et al., 2019; Dienlin and Johannes, 2020), including mental health, social relationships, and quality of life. It was chosen for this theory is that it offers a holistic perspective, helping us understand how specific digital behaviors (Büchi, 2021), like social media sharing, relate to students’ psychological distress and well-being. Moreover, the DWT highlights the interplay between multiple factors (Büchi, 2021), making it well-suited to exploring the complex relationships among different social media sharing behaviors in this study. By applying this theory and incorporating the key variable of perceived support, this paper aims to deeply analyze the mechanisms through which social media sharing behaviors affect student well-being.

Besides, past research has predominantly employed single-variable analysis methods, isolating the impact of individual factors on students’ happiness while neglecting the complex interactions among these factors (Ali-Hassan et al., 2015; Büchi et al., 2019; Zhu et al., 2024). Although this approach helps in understanding the role of specific variables, it falls short in revealing the intricate mechanisms of student happiness under the combined influence of multiple factors (Lee et al., 2022). Therefore, a more comprehensive and in-depth analytical approach is needed to fully uncover the relationship between social media sharing and student happiness (Abowitz and Toole, 2010).

To address these research gaps, this paper aims to conduct in-depth research on the social media sharing behaviors of Chinese college students using analytical methods including Partial Least Squares Structural Equation Modeling (PLS-SEM) and Fuzzy-set Qualitative Comparative Analysis (fsQCA) (Lee et al., 2022). Specifically, this study will explore how different types of social media sharing behaviors relate to students’ psychological distress and well-being, uncovering the complex mechanisms behind these associations. Additionally, drawing from DWT, this paper will offer new perspectives on understanding the impact of social media sharing on student well-being.

The theoretical contributions of this paper are mainly reflected in three key aspects. Firstly, this paper enriches the application scenarios of the DWT (Büchi, 2021; Büchi et al., 2019). By applying it to the study of social media sharing behaviors among Chinese college students, with a special focus on the variable of perceived support, this research not only validates the effectiveness of the DWT in explaining the relationship between social media and student well-being (Büchi et al., 2019; Dienlin and Johannes, 2020; Gui et al., 2017) but also broadens the application scope of the theory (Büchi, 2021; Büchi et al., 2019), offering a useful reference for similar studies. Secondly, this paper reveals the complex mechanisms through which different types of social media sharing behaviors impact student well-being (Utz and Breuer, 2017; Weinstein, 2018). By thoroughly analyzing three types of social media sharing—related to school, social and work—this research finds that these behaviors affect students’ psychological distress and well-being to varying degrees. This insight helps us gain a more comprehensive understanding of the multi-dimensional effects of social media sharing on student well-being, providing a scientific basis for developing targeted intervention strategies (Gerson et al., 2016; Luo and Hancock, 2020; Zhu et al., 2024). Finally, this paper adopts advanced statistical and analytical methods—PLS-SEM and fsQCA, to uncover the complex relationships between social media sharing behaviors and student well-being (Afonso et al., 2018; Lee et al., 2022). The use of these methods not only enhances the accuracy and reliability of the research but also offers methodological insights and inspiration for similar studies.

In summary, by deeply analyzing the relationship between the social media sharing behaviors of Chinese college students and their well-being, this paper aims to provide valuable references and suggestions for educators, policymakers, and students. By revealing the complex mechanisms underlying different types of social media sharing behaviors, it hopes to promote the positive role of social media in enhancing student happiness, contributing to the healthy growth and comprehensive development of students.

## 2 Theoretical framework

### 2.1 Digital well-being theory

Digital well-being theory (DWT) concerns individual’ subjective well-being in a social environment where digital media are omnipresent (Büchi, 2021; Gui et al., 2017). It aims to provide a comprehensive framework for understanding how digital practices, specifically social media sharing, impact individuals’ well-being (Borsboom et al., 2021; Weinstein, 2018). The theory recognizes that the relationship between digital media use and well-being is complex and multifaceted, involving both harms and benefits (Büchi, 2021). At its core, the DWT posits that individuals’ digital practices arise within and shape socio-technical structural conditions. These practices, which encompass various forms of social media sharing such as school-related, social-related, and work-related sharing, lead to often concomitant harms and benefits (Büchi, 2021). For instance, while social media sharing can enhance social connectedness and provide emotional support, it may also lead to privacy concerns, information overload, and psychological distress (Taylor et al., 2023).

The digital well-being framework outlines three pivotal constructs (Büchi, 2021): digital practices, harms/benefits, and well-being. These constructs are interconnected, with digital practices serving as the antecedent that leads to both harms and benefits, which in turn influence individual well-being. The theory emphasizes that the impact of digital practices on well-being is not deterministic but rather contingent upon a variety of factors, including individual differences, social context, and the specific manifestations of digital practices. One of the key contributions of the DWT is its emphasis on the need for empirical research to move beyond simple correlations and focus on developing cumulative science (Vanden Abeele, 2021). This involves identifying plausible causal chains that link specific manifestations of digital practices to individual well-being outcomes. The theory advocates for the use of advanced statistical and analytical methods to uncover these complex relationships (Büchi, 2021). Therefore, the DWT provides a valuable framework for understanding the intricate relationship between social media sharing and individual well-being. By emphasizing the importance of considering both harms and benefits, and the need for rigorous empirical research, the theory offers insights into how we can navigate the digital age in ways that promote overall well-being.

### 2.2 Social media sharing and student well-being

Social media sharing is a significant digital activity, where individuals post academic opinions, social information, and work experiences on social networking platforms. Social media sharing can be categorized into various types, including school-related social media sharing, social-related social media sharing, and work-related social media sharing (Choi and Toma, 2014; Zhu et al., 2024). School-related social media sharing, which includes academic discussions, campus activities, and learning resource sharing (Mao, 2014; Watson et al., 2022), strengthens students’ connections with their school community, enhancing their sense of belonging and academic performance (Mao, 2014), and thus improving overall well-being (Dienlin and Johannes, 2020; Gui et al., 2017). Social-related sharing, focusing on personal life, hobbies, and friends’ updates (Chen et al., 2023; Schmidt et al., 2016), helps students expand social circles and gain emotional support, boosting well-being distress (Dantas et al., 2022). Work-related sharing, significant for students with part-time jobs or internships or projects during their studies (Luo, 2018; Schmidt et al., 2016; Sun et al., 2019), involves professional experiences and industry trends, aiding in building a professional network and boosting achievement and self-confidence, crucial for their academic and career success (Dantas et al., 2022). These different types of sharing behaviors reflect students’ engagement and interests in diverse aspects of their lives and affect their psychological states and well-being through distinct mechanisms.

Social media sharing, as a specific digital behavior, not only reshapes students’ social interactions but also profoundly impacts their mental health and overall well-being (Gerson et al., 2016; Luo and Hancock, 2020), particularly in terms of psychological distress and perceived support (Choi and Toma, 2014). When students face academic pressure, interpersonal tensions, or other psychological distress, over-reliance on social media sharing may exacerbate these problems, reducing their well-being (Dienlin and Johannes, 2020). Moreover, perceived support plays a vital role in moderating the relationship between social media sharing and student well-being (Eagle et al., 2019; Grey et al., 2020). High levels of perceived support from family, friends, or school counselors can buffer the negative effects of psychological distress and enhance the positive impacts of social media sharing on well-being. Conversely, a lack of perceived support may amplify the adverse effects of social media use, leading to further decline in students’ psychological well-being.

Many studies have explored the impact of social media use on individual happiness in both Western and Eastern cultural contexts. In the Western context, Choi and Toma (2014) noted that social sharing on social media influences emotional well-being through interpersonal media patterns; Bekalu et al. (2019) demonstrated that social media use is associated with multiple health indicators, including social well-being, but this relationship varies depending on the method of use. In the Eastern context, Jiang et al. (2023) discovered that online communication and positive psychological capital positively influence the happiness of Chinese college students, with online social support acting as a mediator; Kim and Kim (2017) found that college students’ social media use affects the heterogeneity of their social networks, which in turn impacts their social capital and subjective well-being. These studies collectively indicate that social media plays a positive role in fostering social connections and emotional support, but excessive use can also have negative effects.

However, a cross-cultural study found that the individualism/collectivism framework moderates Chinese participants’ willingness to share and their perceived benefits on social media (Liu and Luo, 2023). Another survey shows that while Chinese youth have information literacy similar to EU youth, they are less skilled in digital content creation and less willing to share actively online (China Mobile Research Institute [CMRI], 2023). The OCCAC Report indicates that people aged 18–29 have higher digital literacy and skills, with college students being the main group engaged in social media sharing (Office of the Central Cyberspace Affairs Commission [OCCAC], 2024). Although studies from various cultural backgrounds have found links between social media use and mental health and well-being, the specific mechanisms and effects may vary across cultures, social contexts, and individual differences. Therefore, this study focuses on Chinese college students to explore how social media sharing behaviors impact their mental health and well-being, aiming to provide new insights and inspiration for related research.

### 2.3 Research hypotheses for PLS-SEM

In this study, to explore the impact of different types of social media sharing behaviors on student well-being, we propose the following research hypotheses based on the DWT, which will be validated using PLS-SEM method.

By sharing school-related content (such as learning experiences and campus activities) on social media, students can strengthen their connections with peers, teachers, and the school, thereby gaining more social support and a sense of belonging, which in turn enhances their well-being (Mao, 2014; Watson et al., 2022). When students share personal life, hobbies, and other social-related content on social media, it helps them expand their social circle, enhance interpersonal relationships, and receive attention and support from others, ultimately boosting their well-being (Sun et al., 2019; Taylor et al., 2023). When students involved in part-time jobs, internships, or projects, sharing work-related content (such as job achievements and professional experiences) on social media allows them to build professional networks (Luo, 2018; Schmidt et al., 2016), showcase their talents, and gain recognition. These positive experiences help alleviate work stress, enhance self-accomplishment, and ultimately improve students’ well-being. Therefore, we propose the following hypotheses:

*H1a: School-related social media sharing has a positive impact on student well-being.*

*H1b: Social-related social media sharing has a positive impact on student well-being.*

*H1c: Work-related social media sharing has a positive impact on student well-being.*

School-related social media sharing can help students feel a stronger sense of belonging and community, which may alleviate psychological distress (Taylor et al., 2023). Through these platforms, students can receive not only academic assistance and resources but also emotional support when facing academic pressures (Lin and Zhan, 2024; Taylor et al., 2023). Social-related social media sharing helps individuals strengthen connections with family and friends, providing essential emotional support and comfort for managing life’s challenges and stress (Taylor et al., 2023). By sharing personal experiences and feelings online, students may experience greater social recognition and acceptance, which can boost self-esteem and social status, thereby reducing psychological distress (Liu et al., 2023). Work-related social media sharing provides students with a sense of achievement and social recognition, supporting their career development by connecting them with resources and opportunities (Luo, 2018). Discussing work-related topics on social media can also serve as a form of stress relief, allowing students to momentarily escape work pressures and potentially easing the psychological distress associated with them (Dantas et al., 2022). Therefore, we propose the following hypotheses:

*H2a: School-related social media sharing has a negative impact on psychological distress.*

*H2b: Social-related social media sharing has a negative impact on psychological distress.*

*H2c: Work-related social media sharing has a negative impact on psychological distress.*

Psychological distress, such as anxiety and depression, is generally negatively correlated with student overall well-being (Deasy et al., 2014; Granieri et al., 2021; Lin and Zhan, 2024). When students experience high levels of psychological distress, they may feel more isolated, helpless, and dissatisfied, thereby reducing their well-being. Therefore, we propose the following hypotheses:

*H3: Psychological distress has a negative impact on student well-being.*

Social media sharing behaviors can have both positive and negative psychological impacts, such as enhancing a sense of belonging or triggering social comparison, which in turn affect students’ psychological states (Gerson et al., 2016; Luo, 2018). Additionally, students’ levels of psychological distress influence how they interpret and react to social media content, indirectly impacting their well-being. For instance, students experiencing high psychological distress may feel more stressed by social media sharing, whereas those with low psychological distress may derive more positive emotions from it (Evers et al., 2020; Taylor et al., 2023). Therefore, we propose the following hypotheses:

*H4a: Psychological distress plays a mediating role between school-related social media sharing and student well-being.*

*H4b: Psychological distress plays a mediating role between social-related social media sharing and student well-being.*

*H4c: Psychological distress plays a mediating role between work-related social media sharing and student well-being.*

We hypothesize that when students experience psychological distress, the support they perceive from family, friends, teachers, or society will mitigate its negative impact on their well-being (Danielsen et al., 2010; Eagle et al., 2019), potentially even transforming it into positive psychological support that enhances their well-being to some extent. Psychological distress is an inevitable part of student life, often arising from academic pressures, interpersonal relationships, future planning, and other challenges (Dyrbye et al., 2006). In these situations, external support serves as a crucial resource, helping students cope with difficulties. Moreover, perceived support is not just an objective resource; it is also a subjective perception that reflects students’ trust and reliance on their social surroundings and relationships (AL-Abrrow, 2022; Chen and Eyoun, 2021; Hakimzadeh et al., 2016). When students feel adequately supported, they are more likely to face challenges with a positive mindset, reducing the negative effects of psychological distress on their well-being. Based on these insights, we propose the following hypotheses:

*H5: Perceived support moderates the relationship between psychological distress and student well-being.*

### 2.4 Research propositions for fsQCA

As previously mentioned, social media sharing behaviors encompass various types such as school-related, social-related, and work-related sharing, each potentially contributing to students’ well-being in distinct ways (Choi and Toma, 2014; Zhu et al., 2024). To gain a deeper understanding of the complex interplay between these factors and their combined influence on students’ happiness, fsQCA analysis becomes indispensable. fsQCA allows for the examination of multiple conditions that may contribute to an outcome and identifies the specific configurations (combinations) of these conditions that are necessary or sufficient for the outcome to occur (Lee et al., 2022).

Firstly, student well-being is a multidimensional and complex concept, shaped by interactions among factors such as school-related, social-related, and work-related social media sharing, psychological distress, and perceived support. This complexity highlights the need to consider the diverse experiences and needs of different students when working to enhance their well-being (Lin and Zhan, 2024). Secondly, individual differences in personality, interests, social skills, and coping strategies lead to varied responses to social media sharing behaviors and differing levels of resilience to psychological distress. This diversity calls for a tailored approach, rather than a one-size-fits-all strategy, when addressing students’ well-being (Kim and Kim, 2017). Finally, the presence of complexity and individual differences suggests that multiple combinations of conditions can effectively enhance student well-being (Manago et al., 2012). Educators, policymakers, and students must flexibly adjust and optimize these conditions based on real-world circumstances and individual needs, as no single optimal configuration applies universally. Exploring and adapting various effective combinations is essential for promoting student well-being.

*P1: There is no single optimal combination of school-related, social-related, and work-related social media sharing, psychological distress, and perceived support that leads to high student well-being; instead, multiple equally effective configurations of these factors can achieve the same outcome.*

No single condition or factor is sufficient to determine students’ well-being; rather, it is the specific combination of multiple conditions that has a significant impact (Lee et al., 2022). For example, in one configuration associated with high well-being, students may exhibit high frequencies of school-related social media sharing, moderate levels of social-related sharing, low psychological distress, and high perceived support. In this combination, each condition interacts with the others, collectively contributing to high well-being. In another equally effective configuration, however, school-related sharing may occur less frequently, while social-related sharing is more frequent; psychological distress remains low, and perceived support is strong. Although this combination differs from the first in specific conditions, it achieves the same outcome of high well-being.

*P2: Depending on their combination with other factors, the same conditions may either be present or absent in different configurations that contribute to high student well-being.*

Ultimately, this study posits the following proposition, which links the findings from PLS-SEM and fsQCA (Lee et al., 2022).

*P3: Configurations leading to high student well-being will necessitate the presence of core elements identified through PLS-SEM.*

## 3 Methods

### 3.1 Questionnaire design

**Figure 1:**
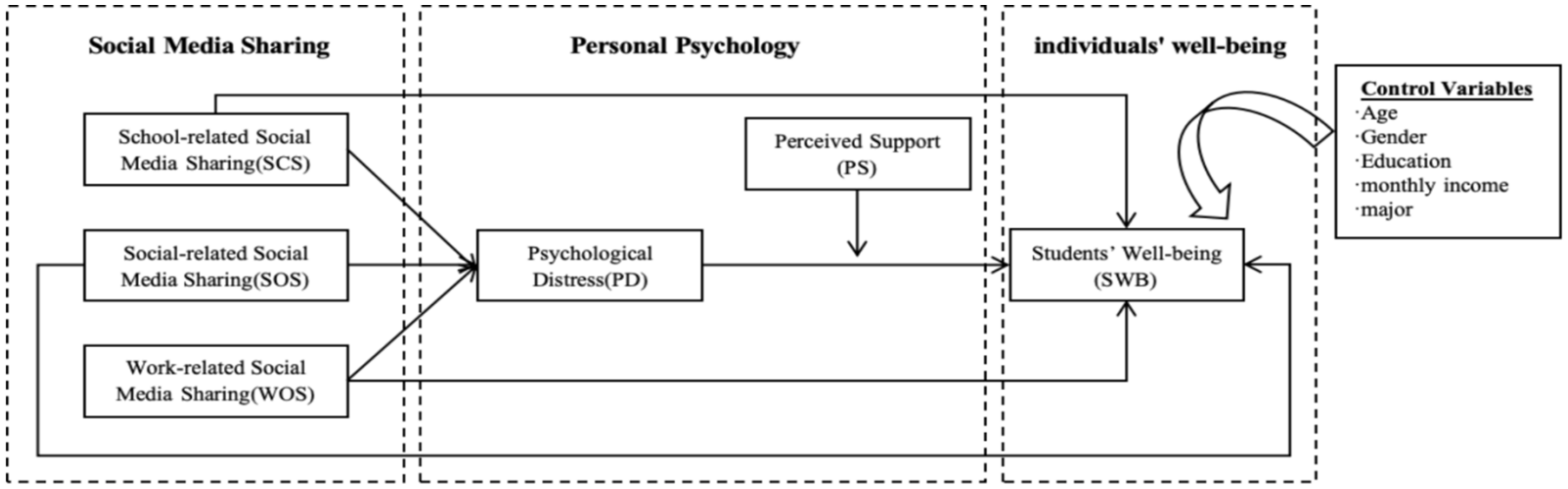
Research framework.

This study designed two-part questionnaire. The first part comprises questions aimed at gathering demographic information from respondents. The second part includes 18 items used to measure the six variables depicted in Figure 1. The scale for measuring school-related social media sharing (Mao, 2014; Watson et al., 2022), social-related social media sharing (Oksa et al., 2021; Zhang et al., 2019), work-related social media sharing (Luo, 2018; Schmidt et al., 2016), psychological distress (Deasy et al., 2014; Lin and Zhan, 2024), perceived support (AL-Abrrow, 2022; Eagle et al., 2019), and student well-being (Gerson et al., 2016; Vanden Abeele, 2021) have excellent reliability and validity in previous studies, making them suitable for this measurement. The questionnaire was designed according to the Likert-7 scale, with responses ranging from ‘1 – Strongly Disagree’ to ‘7 – Strongly Agree’, in conjunction with the contexts of social media sharing. Based on pilot tests by researchers, the scale should take 1–5 min to complete, and participants will be advised that it will not exceed 5 min. Additionally, a screening question was included in the scale to determine whether participants use social media. Only those who confirm using social media can proceed; others are directed to exit the survey.

### 3.2 Data collection

This study utilized convenience sampling, with data collected through an online survey targeting Chinese college students. Participants were recruited using snowball sampling via a WeChat group. Initially, undergraduate and graduate students from two public universities, one private university, and other institutions were invited to join the group. They were then encouraged to invite their classmates and acquaintances to participate. Convenience and snowball sampling may limit the generalizability of the results. Random sampling is recommended in future studies. In total, we recruited nearly 900 college students as participants., each of whom provided their phone numbers and social media account details to facilitate follow-up communications and data collection. Additionally, this study has received ethical approval from the ethics committee of Jinan University (JNU-EC-2024-0015). All collected information will be anonymized to prevent privacy breaches, and participants will be fully informed of these measures.

Drawing on past research, we recognized that collecting data at multiple time points could help reduce the risk of common method bias (Zhu et al., 2024). Consequently, we gathered data at two separate time points. At time point 1, participants in the WeChat group completed questionnaires to provide demographic information (gender, age, year in school, major) and details about their social media sharing habits. A month later, at time point 2, we sent a second questionnaire to those who had completed the first one, this time assessing measures of psychological distress, perceived support and student well-being. In total, we received 693 matched datasets. After filtering out invalid responses—such as those with overly consistent or patterned answers—we retained 534 valid responses. Most respondents attended universities in Guangdong, Shanghai, Hunan, Henan, and other provinces and cities across China. The demographic details of the sample are presented in Table 1.

**Table 1:** Respondents’ demographic profiles (*N* = 534).

Item	Category	Frequency	Percentage
Gender	Male	207	38.76%
	Female	327	61.24%
Age	18–25	295	55.24%
	26–30	202	37.83%
	31–35	37	6.93%
Education	Junior college	77	14.42%
	Bachelor	256	47.94%
	Master	144	26.97%
	Doctor	57	10.67%
Major	Philosophical Sciences	107	20.04%
	Economic Sciences	97	18.16%
	Law	21	3.93%
	Education	35	6.55%
	Literature	15	2.81%
	History	35	6.55%
	Natural Science	143	26.78%
	Engineering	81	15.17%
Monthly income (RMB)	2,500 or below	286	53.56%
	2,501–5,000	45	8.43%
	5,001–7,500	7	1.31%
	7,501 or above	196	36.70%

### 3.3 Data analysis

#### 3.3.1 Reliability and validity test

For data analysis, we utilized SPSS24.0 and AMOS24.0. As depicted in Table 2, the measurement model encompassing all variables underwent confirmatory factor analysis (CFA). The results indicated a satisfactory fit, with *χ*^2^/df at 3.260 (<5), RMSEA at 0.065 (<0.08), IFI at 0.931 (>0.9), CFI at 0.931 (>0.9), NFI at 0.904 (>0.8), and TLI at 0.918 (>0.9).

**Table 2:** Analysis of the model fitting degree.

Indicators	*χ*^2^/df	NFI	TLI	IFI	CFI	RMSEA
Value	3.26	0.904	0.918	0.931	0.931	0.065
Reference range	<5	>0.80	>0.90	>0.80	>0.80	<0.08

As shown in Table 3, the Cronbach’s *α* values for all variables ranged from 0.607 to 0.821, with all values exceeding 0.70, indicating strong reliability. Additionally, the average variance extracted (AVE) values ranged from 0.525 to 0.585, all surpassing the 0.50 threshold (Afonso et al., 2018), suggesting good convergent validity. Significant correlations were observed among all variables, providing partial support for the research hypotheses. The square roots of the AVEs for each latent variable were greater than the correlation coefficients between latent variables, demonstrating good discriminant validity of the measurement model.

**Table 3:** Reliability and validity test.

Variable	Items	Estimate	Cronbach’s *α*	AVE	CR
SCS	I frequently share academic-related content on social media.	0.797**	0.851	0.525	0.908
	I engage in discussions about school activities on social media platforms.	0.726***			
	I share learning resources and tips with my peers via social media.	0.744*			
SOS	I post updates about my personal life and hobbies on social media.	0.788***	0.868	0.576	0.829
	I interact with friends and family regularly on social media.	0.815**			
	I share inspiring stories or quotes that resonate with me on social media.	0.756***			
WOS	I share my professional achievements and experiences on social media.	0.622**	0.771	0.594	0.913
	I discuss industry trends and job-related topics on social media platforms.	0.630*			
	I connect with professionals in my field through social media.	0.607***			
PD	I often feel anxious or worried.	0.771*	0.807	0.585	0.809
	I struggle with feelings of depression or hopelessness.	0.798***			
	I have difficulty sleeping due to stress or worries.	0.724*			
PS	I feel supported and understood by my family and friends.	0.748**	0.789	0.566	0.795
	I have someone to talk to when I am feeling down.	0.821***			
	I receive encouragement and advice from my peers and mentors.	0.681***			
SWB	I am generally satisfied with my life as a student.	0.716**	0.73	0.526	0.768
	I feel a sense of purpose and fulfillment in my academic endeavors.	0.691***			
	I have a positive outlook on my future and career prospects.	0.766*			

***p < 0.001, **p < 0.01, *p < 0.05, SMS, social media sharing; SCS, school-related social media sharing; SOS, social-related social media sharing; WOS, work-related social media sharing; PD, psychological distress; PS, perceived support; SWB, students’ well-being.

#### 3.3.2 Correlation analysis

To assess discriminant validity, we compared the square roots of the AVEs for each latent variable with the pairwise correlation coefficients between variables (Afonso et al., 2018). As shown in Table 4, all correlation coefficients were significant, and the square roots of the AVEs exceeded the correlation coefficients, indicating strong discriminant validity. Additionally, the HTMT ratio further confirmed the discriminant validity of our model.

**Table 4:** Variable correlations.

Variable	SMS	PD	SWB	PS	M	SD
SMS	0.723				4.011	1.369
PD	0.240	0.765			3.267	1.352
SWB	0.600	0.218	0.752		3.861	1.408
PS	0.174	0.271	0.134	0.725	3.438	1.271

The values on the diagonal are the square root of AVE.

## 4 Results

### 4.1 PLS-SEM results

The structural model (Figure 1) was evaluated using AMOS24.0. The Bias-Corrected Bootstrap method was applied to test mediation effects, with a resampling size of 5,000. Demographic variables—namely gender, age, education level, monthly income, and major—were included as control variables. Table 5 displays the results of all hypothesis tests conducted in this study.

**Table 5:** Main effects test results.

Path	Estimate	S.E.	C.R.	*P*	Support
H1a: SCS → SWB	0.198	0.107	2.010	*	Yes
H1b: SOS → SWB	0.234	0.095	2.094	*	Yes
H1c: WOS → SWB	0.247	0.098	2.063	*	Yes
H2a: SCS → PD	0.364	0.097	2.894	**	No
H2b: SOS → PD	0.366	0.110	3.320	***	No
H2c: WOS → PD	−0.449	0.100	−3.359	***	Yes
H3: PD → SWB	−0.136	0.058	−2.561	**	Yes

***p < 0.001, **p < 0.01, *p < 0.05.

For the main effects, the results showed that school-related (*β* = 0.198, *p* < 0.05), social-related (*β* = 0.234, *p* < 0.05) and work-related social media sharing (*β* = 0.247, *p* < 0.05) all had significant positive effects on students’ well-being. Thus, H1a, H1b and H1c all were supported.

Besides, while work-related social media sharing (*β* = −0.449, *p* < 0.001) had significant negative effects on psychological distress, school-related social media sharing (*β* = 0.364, *p* < 0.01) and social-related social media sharing (*β* = 0.366, *p* < 0.001) all had significant positive effects on psychological distress. Thus, H2c were supported, while H2a and H2b was not supported. Moreover, psychological distress had a significant negative effect on students’ well-being (*β* = −0.136, *p* < 0.01), supporting H3.

Under the mediating effect of psychological distress, the total effect values of school-related, social-related, and work-related social media sharing on students’ well-being are 0.161, 0.157, and 0.253, respectively. The 95% confidence intervals for these effects are (0.038, 0.363), (0.026, 0.353), and (0.070, 0.456). None of these intervals include 0, thereby confirming the significance of the mediating effect. Therefore, H4a, H4b, and H4c were supported (Table 6).

**Table 6:** Mediating effects test results.

Path	Effects			Mediating effects		Support
	Indirect effect	Direct effect	Total effect	Bias-corrected		
				Lower	Upper	
H4a: SCS → PD → SWB	−0.054	0.216	0.161	0.038	0.363	Yes
H4b: SOS → PD → SWB	−0.042	0.199	0.157	0.026	0.353	Yes
H4c: WOS → PD → SWB	0.05	0.203	0.253	0.07	0.456	Yes

**Figure 2:**
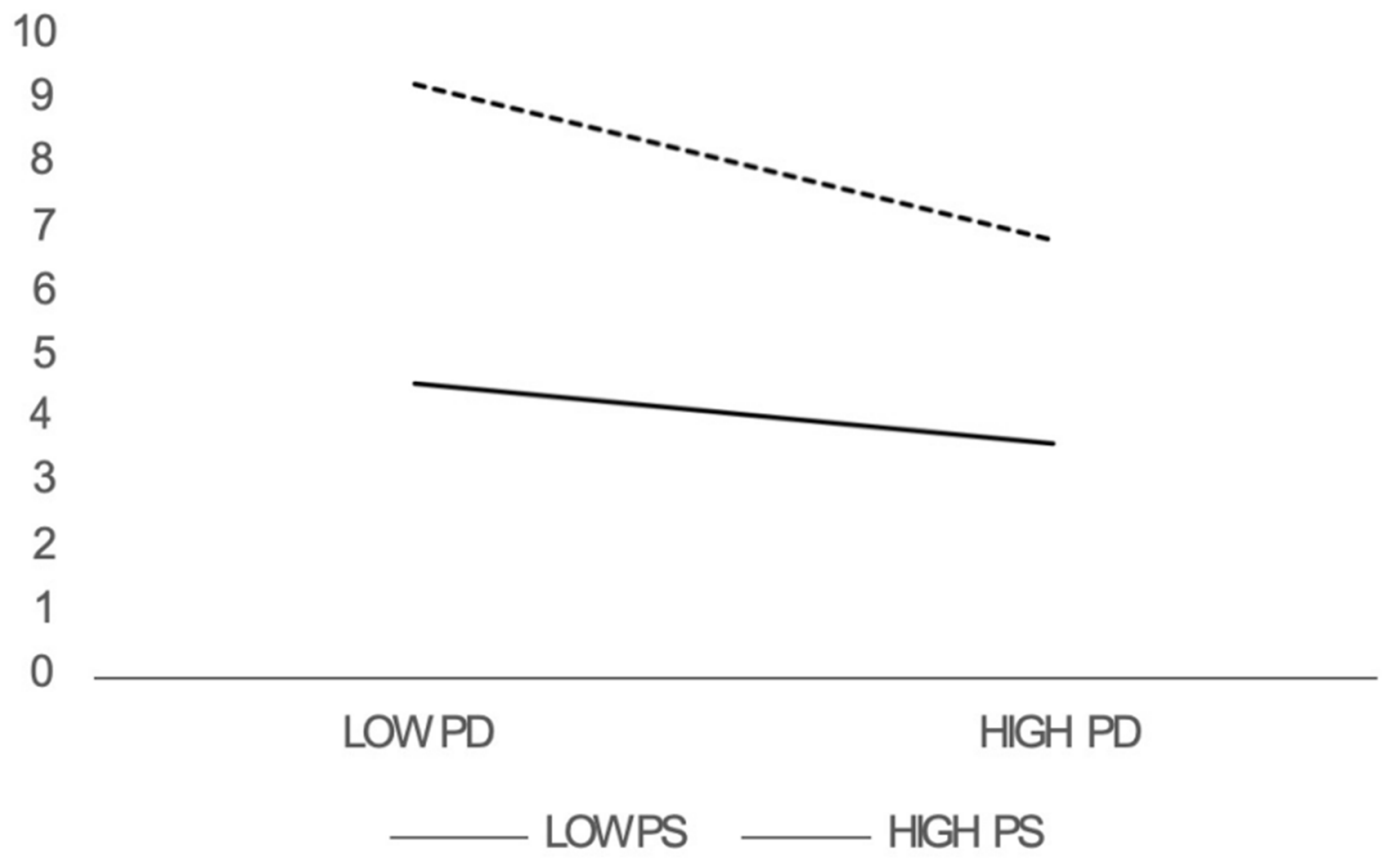
Moderating effects of perceived support.

This study used Model 1 from the PROCESS macro to assess moderation effects. The results showed that the interaction between psychological distress (*β* = 0.136, *p* < 0.001) and perceived support positively predicted student well-being. A simple slope analysis at high (+1 SD) and low (−1 SD) levels of perceived support indicated that psychological distress had a stronger impact on well-being at higher levels of perceived support, demonstrating positive moderation by perceived support. Thus, H5 was supported (see Figure 2).

### 4.2 fsQCA results

In the preceding quantitative analysis based on structural equation modeling (SEM), we verified the direct effects and mediating mechanisms among social media sharing behaviors, psychological distress, and well-being, initially uncovering the statistical associations between variables. However, the SEM method, by its nature, remains a correlation-based analytical framework, and its net effect thinking mode struggles to fully deconstruct the equifinality paths arising from the concurrent effects of multiple conditions in complex social phenomena. To overcome this limitation, this study further introduces fsQCA, aiming to identify multivariate condition combinations driving high well-being through a configurational perspective. This approach complements the SEM results methodologically, constructing a more holistic and ecological explanation framework for the effects of social media use.

This paper conducts an in-depth configurational analysis of five antecedent factors: school-related social media sharing, social-related social media sharing, work-related social media sharing, psychological distress and perceived support. Following widely accepted academic standards, the consistency threshold was set at 0.8, with an additional PRI (Proportional Reduction in Inconsistency) threshold of 0.75 for validity verification (Lee et al., 2022; Wu and Zhou, 2023). Subsequently, the standard analysis process was carried out using fsQCA3.0 software, resulting in complex, parsimonious, and intermediate solutions.

Based on the computational results of these three solutions, the paper selects the intermediate and parsimonious solutions to distinguish between core and peripheral conditions in the configurations (Khan et al., 2022). Core conditions, marked with the symbol “●,” refer to those present in both the parsimonious and intermediate solutions, representing essential elements in the configurational path. Peripheral conditions, indicated by the symbol “○,” appear only in the intermediate solution, suggesting their relatively minor influence on the configuration. When a conditional variable is presented in the form of “~,” it signifies the absence of that variable in the configurational path, marked as “⊗.” If a variable has no impact on the final outcome, it is left blank. Ultimately, the analysis results of the configurational paths are presented in detail in Table 7.

**Table 7:** Configurations for high students’ well-being.

Antecedent factors	Path 1	Path 2	Path 3
School-related social media sharing	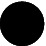		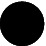
Social-related social media sharing	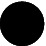	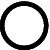	
Work-related social media sharing		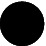	
Psychological distress	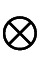	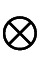	
Perceived support		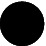	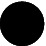
Consistency	0.896487	0.903652	0.903557
Raw coverage	0.522717	0.560358	0.499221
Unique coverage	0.065586	0.116811	0.05052
Solution coverage	0.600119		
Solution consistency	0.906311		

Note: 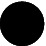 core conditions; 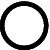 peripheral conditions; 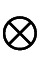 no path.

Table 7 showcases three distinct configurational paths, each with a consistency level exceeding 0.9 (specifically, 0.896487, 0.903652, 0.903557), well above the 0.75 threshold. This indicates that all three paths effectively explain the mechanism underlying users’ continuous usage intentions. Furthermore, the overall solution exhibits a consistency of 0.906311, also surpassing the 0.75 threshold, demonstrating the robustness of the solution in terms of consistency. Furthermore, the overall solution manifests a coverage of 0.600119, implying that these paths account for 60.01% of the factors influencing high students’ well-being. Therefore, the coverage of the overall solution is considered good.

## 5 Conclusion and implications

### 5.1 Discussion of the results

This study focuses on Chinese college students within a collectivist cultural context. The sample’s gender, age, and monthly income distributions are representative of current Chinese college students. However, due to the use of convenience sampling, the educational background and professional fields of the sample, while diverse, exhibit an unbalanced distribution. This paper examined the impact of various social media sharing behaviors (school-related, social-related, and work-related) on the psychological distress and well-being of Chinese college students using two methods: PLS-SEM and fsQCA. Below is a detailed discussion of the research findings.

The results showed that school-related and social-related social media sharing had a significant positive effect on students’ well-being (supporting H1a and H1b), aligning with prior studies (Zhao, 2021; Kysnes et al., 2022). For students’ growth in university, Information related to school and social interaction is critical. Disseminating this information assists students in staying updated on campus developments, expanding their learning resources and social networks, and gaining a sense of control and belonging (Meeus et al., 2022; Smith et al., 2021; Dennen et al., 2024), all of which help to maintain their well-being. However, social media sharing related to school and social interactions had a significant positive impact on psychological distress, which was inconsistent with our initial hypothesis (against H2a and H2b). This finding suggests that while these types of sharing may enhance students’ happiness, they can also inadvertently elevate their psychological stress (He et al., 2024; Yoosefi et al., 2025). Possible reasons for this effect include peer comparison, exposure to negative content, or excessive engagement that distracts from academic and personal responsibilities. Potential reasons encompass peer comparison, exposure to negative content, and excessive engagement that diverts attention from academic and personal duties (Lim and Tan, 2024; Yang et al., 2025).

As expected, work-related social media sharing showed a significant negative effect on psychological distress and a positive impact on happiness (supporting H1c and H2c), which was consistent with previous studies (Yang et al., 2025; Yoosefi et al., 2025). Work-related social media information is relatively inaccessible to students. The sharing of such information can help students clarify their career goals, discover interests, and identify career directions. This accumulates career capital, completing their transition from student life to societal roles (Chan et al., 2018; Wu et al., 2022), and enhancing overall well-being. Moreover, this process helps college students experience social recognition and reduces their stress and anxiety. Psychological distress significantly reduces happiness (supporting H3), which is consistent with previous research findings (Shen et al., 2024). Psychological distress can trigger anxiety and depression in students, making it a key factor in affecting their mental health and overall well-being.

Psychological distress mediates the relationship between social media sharing and well-being (supporting H4a, H4b, and H4c). Social media sharing related to school and social interaction can enhance students’ happiness, yet it also reduces happiness by increasing their psychological distress. These findings, though appearing inconsistent, actually prove the double-edged sword effect of social media mentioned in previous studies (Manago et al., 2020; Kross et al., 2021). The impact of social media sharing on individual happiness is extremely intricate, often being influenced by students’ characteristics and usage frequency (Alphenaar et al., 2025; Ye and Ho, 2025). In addition, work-related social media sharing can reduce students’ psychological distress and thus maintain their happiness. Compared to the other two types of social media information, work-related information is a scarce resource. It meets the long-term developmental needs of college students, aids them in life planning, and enhances their happiness (Vranken and Vandenbosch, 2022). This is a key reason for the differing impacts of the three types of social media sharing.

Perceived support also plays an important role in moderating the link between psychological distress and well-being (supporting H5), showing that when students perceive higher levels of support, the negative impact of psychological distress on well-being is reduced. From an external perspective, students with high perceived support are often adept at acquiring, accumulating, and utilizing social resources. Positive external support can help mitigate the negative effects of psychological distress (Zhao et al., 2022). Internally, perceived support helps students develop a positive cognitive style, making them more likely to view psychological distress as a temporary challenge rather than an insurmountable obstacle, which can enhance their overall well-being (Huang, 2016). This suggests that perceived support can act as a buffer, helping students better manage psychological distress and maintain a higher level of well-being (Vranken and Vandenbosch, 2022).

### 5.2 Theoretical implications

This research offers significant contributions to the literature on social media psychology and DWT.

Firstly, extended the application scope of DWT (Büchi, 2021; Büchi et al., 2019). It is the first to apply DWT to analyze how different types of social media sharing behaviors (school-related, social-related, and work-related) among Chinese college students impact their psychological well-being (Büchi et al., 2019; Dienlin and Johannes, 2020; Gui et al., 2017). Empirical validation demonstrates DWT’s effectiveness in explaining this relationship and extends its application to new contexts, providing valuable references for future studies (Büchi et al., 2019; Vanden Abeele, 2021).

Secondly, uncovered the complex mechanisms of social media sharing behaviors (Utz and Breuer, 2017; Weinstein, 2018). Through the comprehensive use of PLS-SEM and fsQCA, this study explores the intricate mechanisms by which different types of social media sharing behaviors affect student well-being (Gerson et al., 2016; Luo and Hancock, 2020; Zhu et al., 2024). The findings highlight significant variations in how these sharing behaviors influence well-being, deepening our understanding of the impacts of social media usage and offering a scientific foundation for developing targeted interventions.

Lastly, promoted the use of multivariate analysis methods in social media research. By adopting PLS-SEM and fsQCA, this study overcomes the limitations of traditional single-variable analysis approaches (Afonso et al., 2018; Lee et al., 2022). It uncovers the complex interactions between social media sharing behaviors and student well-being. The application of these integrated analysis methods not only improve research accuracy and reliability but also provide new methodological insights for future social media studies (Deasy et al., 2014; Mao, 2014), advancing the field and promoting a more comprehensive understanding of the phenomenon.

### 5.3 Practical implications

The findings of this study offer valuable practical guidance on the social media usage behaviors of Chinese college students and their impact on well-being.

Firstly, the results guide educators and students in optimizing their social media usage strategies. Educators can encourage students to actively participate in school-related social media activities, such as academic discussions and campus event sharing, to enhance their sense of belonging and academic support. At the same time, students should be advised to engage in social-related sharing moderately to expand their social circles and receive emotional support. For students involved in part-time jobs or internships, appropriate sharing of work-related content can help build professional networks and showcase talents. Additionally, educators should be mindful of students’ psychological stress and provide timely psychological support and counseling.

Secondly, this study underscores the importance of strengthening mental health education and services. Given the negative impact of psychological distress on student well-being, it emphasizes the need for universities to strengthen mental health education. Regular mental health workshops, lectures, and counseling sessions can help students manage emotional issues. Schools can also establish mental health support systems, such as counseling hotlines and online platforms, to provide timely assistance and interventions. Furthermore, educators should encourage students to seek mental health services and reduce the stigma surrounding mental health issues.

Lastly, the study highlights the critical role of promoting collaboration between family, school, and social support systems to enhance student well-being. Parents should prioritize their children’s mental health, maintain open communication, and offer emotional support and practical help. Schools should strengthen cooperation with parents to jointly monitor students’ psychological well-being and address any issues promptly. Additionally, schools can also invite social professionals to participate in mental health education, providing students with more diversified forms of support. This collaborative approach can effectively alleviate students’ psychological distress and improve their overall well-being.

### 5.4 Limitations and future research suggestions

Although this study has made meaningful contributions to understanding the impact of social media sharing behavior on the mental health and well-being of Chinese college students, several limitations and suggestions for future research remain. One limitation is the narrow sample scope, as it primarily comprises Chinese college students, which may restrict the generalizability of the findings. Students from diverse cultural and social backgrounds might have varying social media usage patterns, mental health experiences, and perceptions of happiness. Future research could expand the sample to include students from various countries and cultures to test the applicability of these findings across contexts (Bailin, 1993). Another limitation is the use of a single research method. While the two-round data collection introduced a temporal dimension to the study, the data rely entirely on self-reports from students, risking self-reporting bias. Several factors may compromise the accuracy of responses regarding social media use, psychological distress, and well-being, such as memory failings, social desirability bias, and personal subjective perceptions. To enhance the objectivity and precision of future research, researchers could combine multiple data sources and methods (Wang et al., 2025), such as direct observation, interviews, or social media usage data from platforms (Ahmed et al., 2021; Utz and Breuer, 2017).

## Data Availability

The original contributions presented in the study are included in the article/supplementary material, further inquiries can be directed to the corresponding authors.
