# Correction: Social media sharing, psychological distress, and student well-being: a PLS-SEM and fsQCA analysis of Chinese college students

**DOI:** 10.3389/fpsyg.2025.1689669

**Published:** 2025-09-03

**Authors:** Bo Shu, Gaozhi Dong, Fang Su, Zheng Wang

**Affiliations:** ^1^School of Management, Jinan University, Guangzhou, China; ^2^Faculty of Health and Wellness, City University of Macau, Macau, Macao SAR, China; ^3^Department of Public Administration, Jiangxi Administration Institute, Nanchang, China

**Keywords:** social media sharing, psychological distress, students well-being, digital well-being theory, PLS-SEM, fsQCA

In the published article, there was a mistake in the second author's name. Author Gaozhi Dong was erroneously spelled as “Zhigao Dong.”

The original version of this article has been updated.

